# Malformation of a Mesocolon as a Cause of Colic in an Arabian Foal

**DOI:** 10.3390/vetsci8090193

**Published:** 2021-09-13

**Authors:** Bernard Turek, Elżbieta Stefanik, Natalia Kozłowska, Olga Drewnowska-Szczepakowska, Kamil Górski, Julia Mickiewicz

**Affiliations:** Department of Large Animal Diseases and Clinic, Institute of Veterinary Medicine, Warsaw University of Life Sciences, Nowoursynowska 100, 02-797 Warsaw, Poland; natalia_kozlowska@sggw.edu.pl (N.K.); vet.olgadrewnowska@gmail.com (O.D.-S.); kamil_gorski@sggw.edu.pl (K.G.); julia_mickiewicz@sggw.edu.pl (J.M.)

**Keywords:** congenital malformation, mesocolon, foal, colic

## Abstract

This paper describes a case of partial lack of the mesocolon in a 7-month-old colt. The foal was referred to the hospital with clinical signs of severe abdominal distension of a few hours duration. Because analgesics did not relieve pain, the foal remained uncomfortable, and distension of the abdomen increased; an exploratory laparotomy was performed under general anaesthesia in dorsal recumbency. The final diagnosis was confirmed intraoperatively. During exploration of the abdominal cavity, other problems like right dorsal displacement and torsion of the colon were recognized. Correction of all problems was completed, and the mesentery was sutured. Recovery from anaesthesia was uneventful. The foal was recovering well a few months after surgery, and the owner did not complain about the results of the treatment.

## 1. Introduction

Abdominal pain, described as colic, is a relatively common problem in equine practice [1]. The most common causes in foals include meconium impaction, infectious and non-infectious enteritis, functional or mechanical obstruction, intussusception, gastric and duodenal ulceration, rupture of the urinary bladder, peritonitis and complicated hernias [2,3]. Nevertheless, rare congenital abnormalities should also be considered in the differential diagnosis of colic in foals [4,5,6,7].

Congenital disorders are reported in approximately 3.5% of newborn foals, of which only 3.1% are gastrointestinal anomalies. The most frequently described abnormality of this type is intestinal atresia [7,8,9]. Because mesentery is involved in supplying the intestines with blood through the mesenteric arteries, one of the mechanisms of congenital bowel defects is the disorder of blood supply in the fetal period caused by abnormalities of the mesentery [10]. The complex mechanism of mesentery formation during fetal development and its direct influence on intestinal development may result in a variety of malformations [6,11,12,13]. However, cases have been described in which the abnormal development of the mesentery was an isolated abnormality, and the intestine developed properly due to the formation of collateral circulation [4,14,15].

In the diagnosis of colic in foals, a rectal examination is not applicable due to their small body size. However, balloting, abdominal palpation, radiography of the abdomen and ultrasound examination may be helpful [2,16]. Sometimes, as in the described case, laparotomy remains the only diagnostic option.

The mesenteric defects predispose the foal to increased intestinal mobility within the peritoneal cavity, which may lead to subsequent development of various types of colic [7,10,14,15,17]. The described case of congenital defect of the mesenteric colon is an example of a rare developmental disorder that can cause colic in foals, not previously described thoroughly in the literature.

## 2. Materials and Methods

### 2.1. Case Presentation

A 7-month-old purebred Arabian foal was referred to the clinic because of non-resolving colic symptoms. The first signs of abdominal discomfort appeared several hours earlier and were gradually progressing.

The foal was restless in the stall, lying down and rolling. The abdominal distension was noted along with significant bloating in the right flank region. Clinical examination showed dry, pale-pink mucose membranes. Capillary refill time was prolonged to 4 s. The pulse rate was elevated to 90 beats per minute, and the respiratory rate reached 32 breaths per minute. Body temperature was within normal physiological limits (37.8 °C). Auscultation of the abdominal cavity revealed weakened peristaltic murmurs and increased amounts of gas in the right and left flank region.

Due to the size of the animal, a rectal examination was omitted. After the placement of a nasogastric tube, 8 L of reflux was evacuated. An ultrasound examination revealed a large amount of free fluid in the abdominal cavity. In the lower part of the left flank, bloated loops of small intestines were visualized, and in the right flank, the large intestine was observed to have a significantly thickened wall. External jugular vein catheter was placed, the blood sample was collected, and fluids (Ringer with lactate, 40% dextrose solution, and amino-acid solution) were administered. Blood count showed dehydration (RBC 15.21 × 10^12^/L, HGB 227 g/L, HCT 60.5%), and total protein was elevated (7.8 g/dL). Because of no improvement after analgesic treatment and the patient’s deteriorating condition, surgical intervention was chosen.

### 2.2. Surgery and Postoperative Treatment

**Figure 1:**
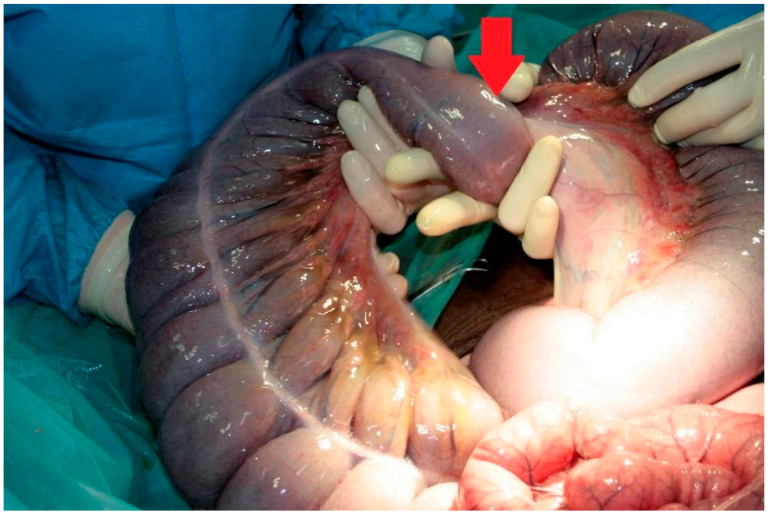
Torsion of the left colon (red arrow).

**Figure 2:**
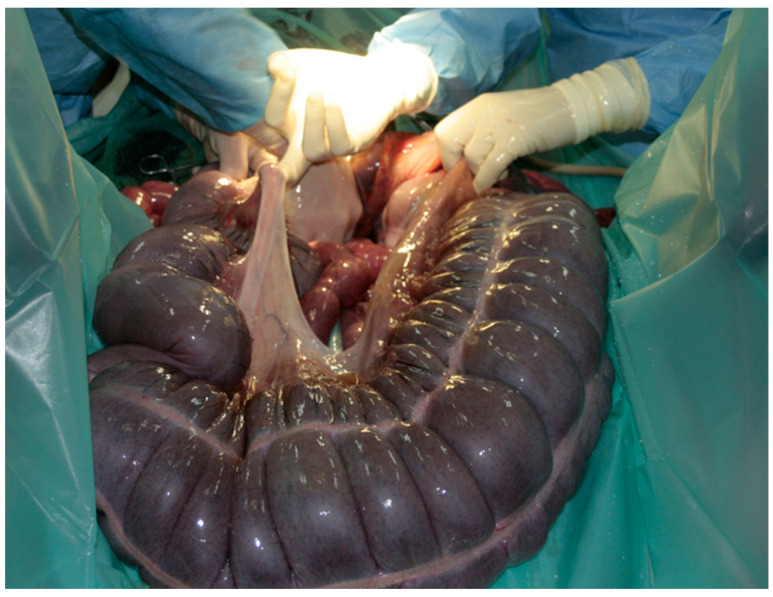
Defect of the left mesentery (after removal of the small intestine loop and transverse colon).

**Figure 3:**
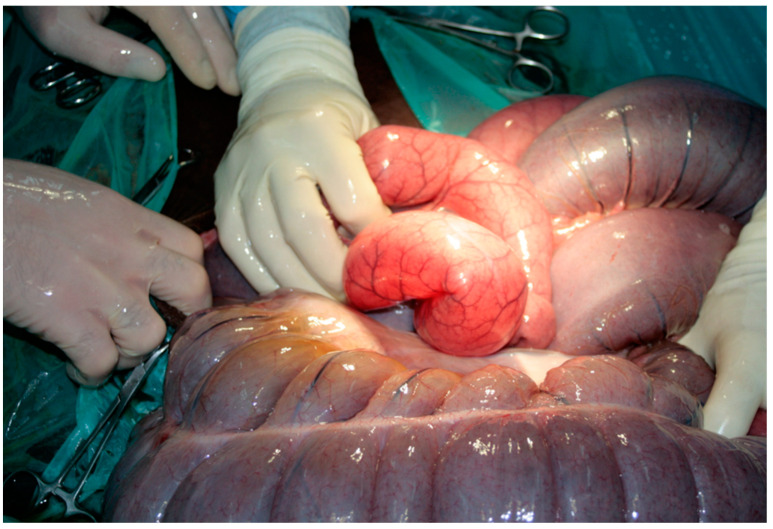
Bloated loops of the small intestines are visible in the mesenteric defect.

Premedication of the foal was performed with diazepam at a dose of 0.25 mg/kg; the induction was performed with ketamine at a dose of 2 mg/kg. Anaesthesia was maintained with isoflurane (induction 5%, maintenance 2–3%). After the foal was placed in dorsal recumbency, a median laparotomy was performed. A thirty-centimetre incision was performed in the white line through the umbilicus in both the caudal and cranial directions. Upon inspection of the abdominal cavity, the right dorsal displacement of the large colon with torsion was identified (Figure 1). The wall of the colon was thickened and cyanotic as a result of impaired blood circulation, with no obvious areas of necrosis. The dorsal mesentery was underdeveloped (Figure 2), as a result of which there was a free space between the dorsal and ventral colon with loops of the small intestines and small colon (Figure 3).

**Figure 4:**
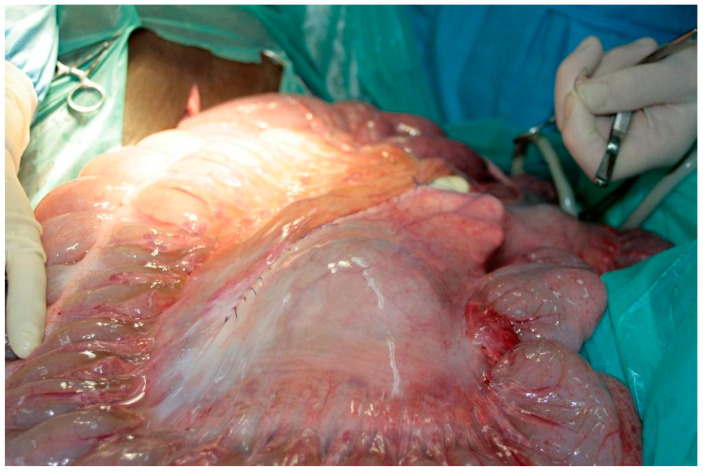
Solving the primary problem by suturing abnormally formed mesentery in the colon.

After the restoration of the physiological position of the colon, the return of its motility and normal colouration were observed. The edges of the mesentery were refreshed and then sutured with PGA 2/0, using a simple continuous suture (Figure 4). The recovery from anaesthesia was uneventful. For analgesic treatment, flunixin meglumine (1.1 mg/kg, i.v.) was used. The implemented antibiotic therapy consisted of ceftiofur sodium at 5 mg/kg i.m. and gentamicin at 6.6 mg/kg i.v. for the following 5 days. During clinical examination, no other signs of congenital abnormalities were found. The horse returned home 2 weeks after the surgery. According to the authors’ knowledge, no recurrences of the disease were recorded within 2 years.

## 3. Discussion

According to the latest reports, mesentery (*mesenterium*) has been classified as a separate organ of significant importance in gastroenterology [18,19,20]. Its structure has been proven to be homogeneous, continuous and unchanged, regardless of which organ it is adjacent to [13]. At the beginning of its development, the primary gut is suspended to the abdominal wall by the mesoderm, which develops into dorsal and ventral mesentery. The increase in length and further development of the gastrointestinal tract during the prenatal period results in elongation of the dorsal mesentery, in the middle of which the mesenteric artery (*arteria mesenterica cranialis*) runs [21]. The ventral mesentery regresses caudally from the duodenum and cranially from the rectum. Regression of the ventral mesentery allows further development of the intestine through a complex mechanism of its increase in length and rotation around the cranial mesenteric artery [6]. The rotation process is crucial for the final positioning of the intestines in the peritoneal cavity. It is during this stage that the final mesenteric attachments with the abdominal wall are formed [10]. Postnatally, the superior mesentery extends from the distal end of the oesophagus, through the stomach to the duodenum, and forms the lesser omentum and ligaments of the liver, whereas the dorsal mesentery runs along the entire length of the peritoneum [15]. The pathogenesis of mesenteric malformations is still not well understood. Developmental abnormalities may occur at any stage of mesenteric formation and affect different segments of the mesentery. These defects may affect the entire length of the mesentery (basal defects) or only some of its segments (segmental defects) [10,15]. The possible causes of mesenteric abnormalities are mesenteric atresia during prenatal development or developmental restriction of mesenteric blood supply caused by its too rapid elongation. Congenital mesenteric abnormalities have also been reported to frequently coexist with other gastrointestinal anomalies, particularly small bowel atresia. Some reports suggest a genetic aetiology of mesenteric defects and an association with Hirschprung’s disease or cystic fibrosis in humans [22].

**Figure 5:**
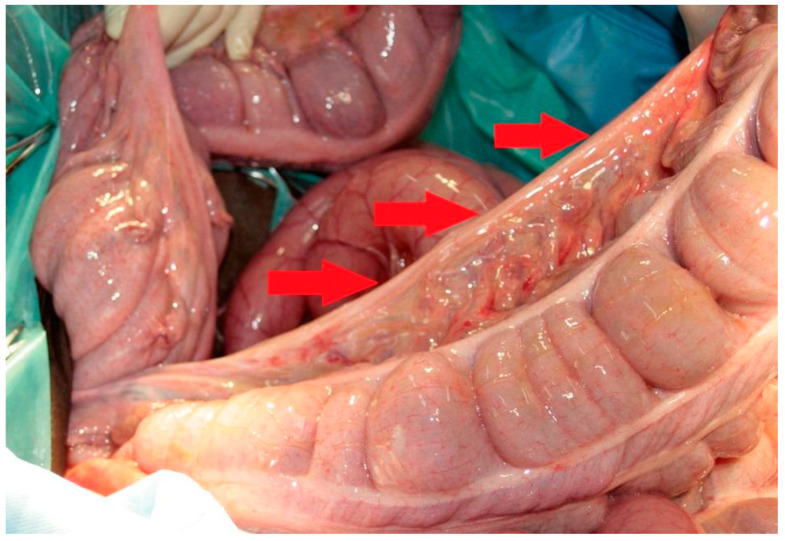
Thickened edge of the mesentery (red arrows).

In human medicine, cases of internal hernias related to the mesenteric defect at its different segments have been described. The incidence of this anomaly among children is estimated at 0.2–0.9% [23]. Congenital defects most often affect the mesentery of the small intestine with a typical diameter of 2–3 cm, located close to the Treiz ligament or the ileocecal valve, which most often leads to entrapment of small intestine loops or small colon. The clinical symptoms are severely expressed, the diagnosis is nonspecific, and a laparotomy is an optimal solution. Apart from congenital defects, the mesenteric defects may occur as a result of inflammation, trauma or postoperative complications, with petechiae, bleeding or inflammatory lesions being present as a result of vascular damage. The thickened edge of the mesentery has been observed in cases of congenital defects [24]. In the presented case, no features of inflammation or trauma were found. Moreover, the edges at the defect were markedly harder and thickened (Figure 5), clearly indicating a congenital defect. In the described cases available in the literature, the first clinical signs appeared in adulthood [25]. In the case presented here, the first symptoms appeared relatively late, at the age of 7 months. It is important to point out that in horses, the gastrointestinal tract is quite loosely fixed in the abdominal cavity and is, therefore, prone to many disorders. The most likely cause of displacement occurs when horses roll. There is a high probability that also in the presented case, there was a stimulus initiating the displacement, which, in the absence of proper fixation of all portions of the colon, led to very advanced changes. So far, only a few cases of congenital mesenteric defects have been well documented in horses. The described cases have included the abnormal formation of the mesentery of the colon [26], lack of dorsal attachment of the large colon and cecum [14], and type 3 colonic atresia, where the segment of the colon and its mesentery have failed to develop [27,28]. There was also a case in which abnormalities in the formation of the mesentery of the colon were considered to be the cause leading to the formation of the T-shaped colon [6].

Regardless of the identified defect, due to the stabilizing and suspending function of this structure, all its anomalies predispose it to the development of colic related to secondary intestine displacement, torsion and volvulus; it is also not uncommon for the intestines to become strangulated within the gates of the internal hernia [7,10,15,17]. For this reason, the disease is most often diagnosed early in the life of foals as a result of diagnosis of acute colic. These abnormalities are less frequently diagnosed in older horses, in which they may cause recurrent colic [7,26]. It is difficult or even impossible to diagnose this problem before surgery. The mesentery is difficult to visualize in diagnostic imaging methods; only some MRI and CT settings are able to visualize it [29]. Both in the described case and in the available literature, the disorder was diagnosed intraoperatively. The differential diagnosis should consider acquired mesenteric discontinuity, which may be the result of trauma, overextension of the mesentery secondary to other conditions (e.g., ileal impaction) or careless manipulation of the mesentery during surgical procedures. A mesenteric hernia is also found in breeding mares as a result of trauma occurring during parturition [30].

## 4. Conclusions

Congenital anatomical anomalies should always be considered in the differential diagnosis of changes in foals and young horses. The presented case emphasizes the high importance of a quick decision on surgical treatment of diagnostic laparotomy in making a definitive diagnosis and increasing the chances of survival.

## Data Availability

No new data were created or analyzed in this study. Data sharing is not applicable to this article.
